# Dual-Color Live Imaging of Adult Muscle Stem Cells in the Embryonic Tissues *of Drosophila melanogaster*

**DOI:** 10.21769/BioProtoc.4605

**Published:** 2023-02-05

**Authors:** Monika Zmojdzian, Binoj Dhanarajan, Krzysztof Jagla, Rajaguru Aradhya

**Affiliations:** 1 GReD Institute - UMR CNRS 6293 / INSERM U1103 University of Clermont-Auvergne, Clermont-Ferrand, France; 2 School of Biotechnology, Amrita Vishwa Vidyapeetham, Kollam, Kerala, India

**Keywords:** Muscle stem cells, *Drosophila*, Live imaging, Embryo, Time-lapse microscopy, Adult muscle precursors, GFP, mCherry, Peripheral nervous system, 3D image analysis

## Abstract

Adult muscle stem cells (MuSCs) show remarkable capability in repairing injured tissues. Studying MuSCs in suitable model organisms, which show strong homology with vertebrate counterparts, helps in dissecting the mechanisms regulating their behavior. Additionally, ease of handling and access to technological tools make model organisms well suited for studying biological processes that are conserved across species. MuSCs quiescence, proliferation, and migration are regulated by various input of signals from the surrounding tissues that constitute the MuSCs niche. Observing MuSCs along with their niche in vivo through live imaging provides key information on how MuSCs behave in quiescent and activated states. *Drosophila melanogaster* is well known for its genetic tool arsenal and the similarity of its different biological processes with vertebrates. Hence, it is widely used to study different types of stem cells. Gained knowledge could then be extrapolated to the vertebrate/mammalian homologous systems to enhance our knowledge in stem cell fields. In this protocol, we discuss how to perform live cell imaging of *Drosophila* MuSCs, called adult muscle precursors (AMPs) at embryonic stages, using dual-color labelling to visualize both AMPs and the surrounding tissues. This dual-color fluorescent labelling enables the observation of in vivo behavior of two types of cells simultaneously and provides key information on their interactions. The originality of this protocol resides in its biological application to MuSCs and their niche.

## Background

**Figure 1.**
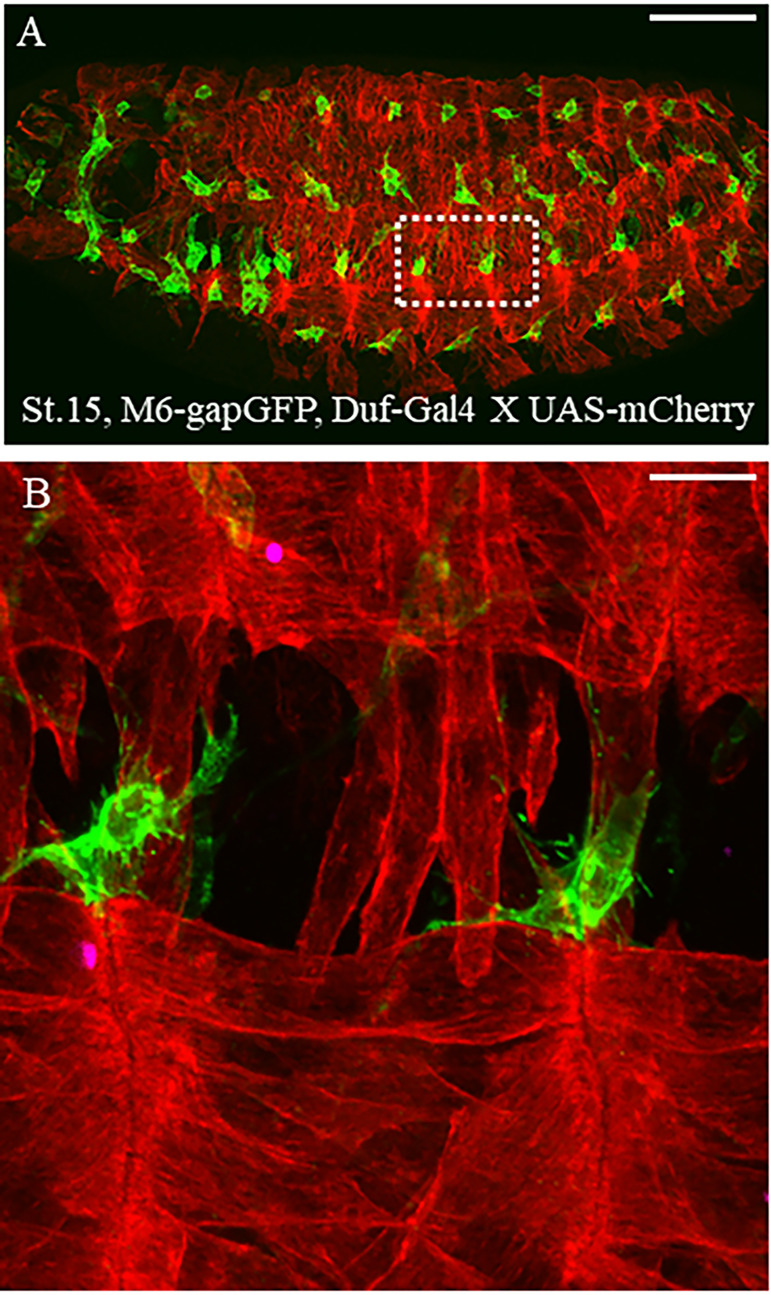
Dual-color live imaging of muscle stem cells and surrounding tissues. (A) Lateral view of stage 15 *Drosophila* M6-GapGFP; Duf-Gal4 X UAS-mCD8-mCherry embryos; AMPs expressing GFP are in green and the differentiated muscles expressing mCherry are in red. Scale bar: 100 μm. (B) Two hemisegments around the lateral muscles and associated AMPs have been magnified to observe the ultrastructure of cytoplasmic extensions protruding from AMPs. Scale bar: 9 μm.

*Drosophila*'s adult muscle precursors (AMPs) are quiescent muscle stem cells specified from the mesodermal lineages during embryonic stage 12 (Bate et al., 1991; Figeac et al., 2010; Aradhya et al., 2015). They form as sibling cells of muscle progenitor cells, giving rise to differentiated skeletal muscle tissue for larval locomotion. However, AMPs remain quiescent and non-differentiated during the embryonic and first parts of the larval life (Aradhya et al., 2015). The ability to maintain their quiescent nature makes AMPs an attractive model to study mechanisms regulating their dormant state, which could be homologous to those that control quiescence of mammalian muscle stem cells (MuSCs) that ensure repair of damaged muscle tissue (Sambasivan and Tajbakhsh, 2015). Through live cell imaging, using a gap-GFP transgene to mark cell membranes, we have previously demonstrated that AMPs display dynamic cellular processes during their quiescent state (Figeac et al., 2010; Aradhya et al., 2015). In contrast to the dot-like pattern of AMPs revealed by antibody staining against the nuclear protein Twist (Bate et al., 1991; Broadie and Bate, 1991; Sellin et al., 2009), our time-lapse live imaging allowed to observe that quiescent AMPs send both filopodia and cellular projections to the neighboring cells (Figeac et al., 2010). This new finding encouraged us to look deeper into the cellular behavior of AMPs through further generation of molecular and genetic tools for dual-color live imaging. Using these new transgenic *Drosophila* lines and in-depth observations through confocal imaging, we showed that AMPs interact with the embryonic muscles and the peripheral nervous system (Aradhya et al., 2015). We were able to visualize how filopodia projected by AMPs find the surrounding muscles, which in turn serve as their niche (Figure 1). These fine cellular structures would not have been discovered in fixed tissue due to the harsh nature of fixative agents. Hence, studying the cellular nature of a given cell type during development through live imaging provides a better resolution of the tissue morphogenesis. Dual-color live imaging allows documenting the dynamic behavior of two types of cells/tissues over time (Video 1). In this article, we describe the detailed protocol for performing dual-color time-lapse live imaging in *Drosophila* embryos using our previously generated molecular tools (Aradhya et al., 2015). The readers can apply this protocol to their own transgene combinations to label other cells of interest.

Though there are other methods to visualize multiple cell types with different colors in *Drosophila*, they require the construction of a fluorescent gene cassette combined with GAL4 drivers, each specific to the cells of interest or unable to label tissues that are different in origin. Additionally, the signal intensity and ability to observe fine cellular structures at embryonic stages are comparatively weaker in the method we have described in this protocol (Boulina et al., 2013, Hadjieconomou et al., 2011, Hampel et al., 2011). The strength of the genetic tool mentioned here lies in combining already known enhancer driver lines, which directly drive the expression of GFP cassette, instead of using a binary expression system such as UAS-GAL4 in the cells of interest. GFP cassette expression through binary systems tends to delay the temporal activity of a given enhancer. Also, restricting the expression of GFP cassette under the regulation of cell type–specific enhancers allows the manipulation of complementary cell types using other transgenes, such as RNA interference lines, against a specific gene by incorporating a separate binary expression system using a simple genetic cross.

## Materials and Reagents

1. Nunc^TM^ Thermanox^TM^ coverslips (Fisher Scientific, catalog number: 174942)
2. Flystuff embryo collection cage-mini, fits 35 mm Petri dishes (Genesee Scientific, catalog number: 59-105)
3. Fisherbrand^TM^ dissecting needle wood (Fisher Scientific, catalog number: 13-820-024)
4. Double-sided tape (Scientific Industries, catalog number: SI1616)
5. Nunc^TM^ cell culture/Petri dishes (Fisher Scientific, catalog number: 12-565-90)
6. Nunc^TM^ square BioAssay dishes (Fisher Scientific, catalog number: 166508)
7. Flystuff mesh basket, small, 3/4 inch inside diameter (Genesee Scientific, catalog number: 46-101)
8. Flystuff Nitex nylon mesh 630 μm, 45 inch wide roll, 1 foot/unit (Genesee Scientific, catalog number: 57-101)
9. Dechorionation chamber, prepared by adding a suitable size of Flystuff nylon mesh to the Flystuff mesh basket
10. Agar powder (Fisher Scientific, catalog number: A10752.22)
11. Sucrose (crystalline/certified ACS) (Fisher Scientific, catalog number: S5-500)
12. Apple/grape juice (any commercial product available from local sources)
13. Yeast granules (any commercial product available from local sources)
14. Methyl 4-hydroxybenzoate, 99% (Fisher Scientific, catalog number: AAA1428930)
15. Sodium hypochlorite solution (commercial bleach), available chlorine 4% (Fisher Scientific, catalog number: Q27908)
16. N-heptane, certified AR for analysis (Fisher Scientific, catalog number: H/0160/15)
17. Halocarbon oil 27 (Sigma-Aldrich, catalog number: H8773
18. Ultra-soft tissues (Kleenex)

**Fly stocks**

1. Duf-Gal4 (a gift from K. Vijayraghavan, NCBS, India)
2. M6-GapGFP [lines were created as part of a previous study (Aradhya et al., 2015)]
3. UAS-mCD8-mCherry (Bloomington Drosophila Stock Center, stock number: BL27391)
  Note: All stocks should be maintained at 25 °C on standard *Drosophila* food medium.

## Equipment

1. *Drosophila* incubator (Percival, catalog number: DR-36VL)
2. P10 micropipette (Eppendorf, Catalog No. 3123000020)
3. Stereo dissecting microscope (Olympus Stereo Microscope System, catalog number: SZX7)
4. Leica TCS SP5 confocal microscope

## Software

1. Imaris (BitPlane), https://imaris.oxinst.com/
2. Fiji (ImageJ), https://imagej.net/software/fiji/downloads

## Procedure

**Figure 2.**
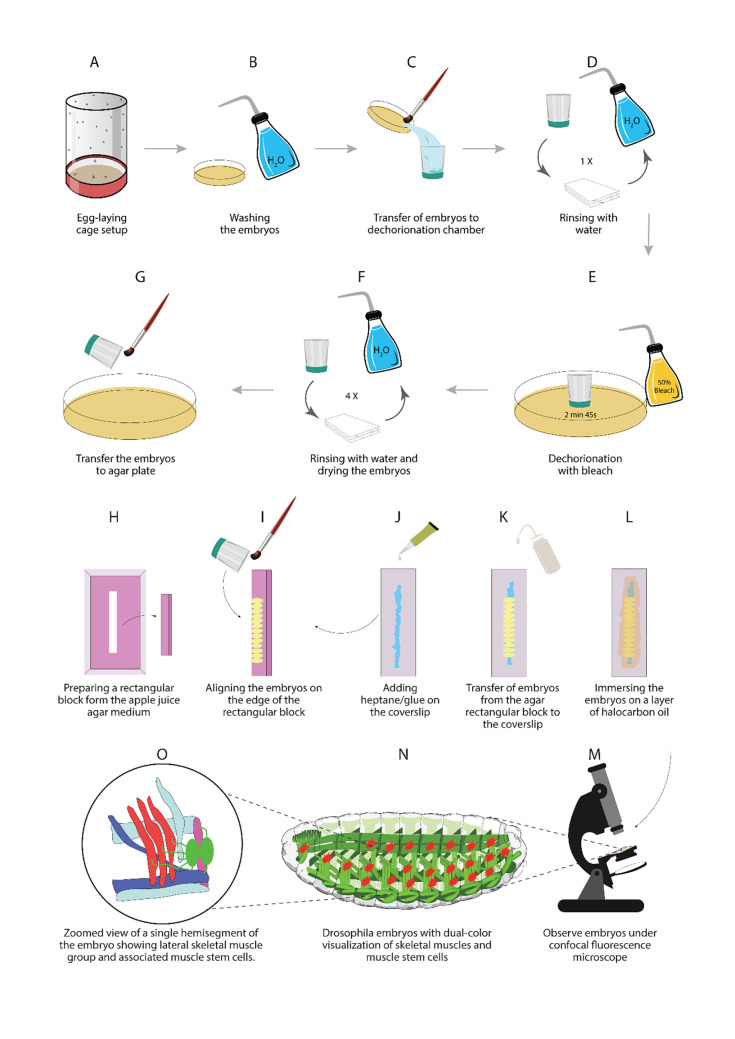
Illustrations of different steps described in this protocol

- ***Drosophila* cage setup, embryo collection, and dechorionation**
  1. Set up a cross between the M6-gapGFP, Duf-Gal4, and UAS-mCD8-mCherry transgenic flies with 40–60 flies in an embryo collection cage of suitable size (Figure 2A).
  2. Prepare apple/grape juice agar medium using methyl 4-hydroxybenzoate according to standard protocols (Cold Spring Harb Protoc, 2011), pour it into 35 mm cell culture dishes, and allow the food medium to solidify at room temperature. The plates with medium can be stored at 4°C for up to two weeks (Figure 2A).
  3. Add a layer of yeast paste to the center of the apple juice agar plate before setting up the cage to stimulate the egg production.
  4. Allow the flies to mate and start laying eggs in the egg-laying cage for two days in a 25°C incubator with a 12:12 h light/dark cycle.
  5. Once the flies are synchronized to the cage, the apple juice agar plates can be changed every 12 h to collect embryos with a developmental time point ranging from 0 to 12 h. Alternatively, collect the egg-laying plates by transferring flies to a new egg collection cage every 2 h and incubate separately in a 25°C *Drosophila* incubator to obtain age-synchronized embryos.
  6. Wash the embryos from the plates with a fine brush into a dechorionation chamber; rinse with water repeatedly to remove any traces of yeast paste (Figure 2B–D).
  7. Dechorionate embryos using 50% commercial bleach with continuous swirling of the dechorionation chamber for up to 2 min, until all embryos look like shiny rice granules. At this step, embryos can be observed under the dissection microscope to ensure proper removal of the chorion membrane (Figure 2E).
  8. Thoroughly rinse the dechorionation chamber with distilled water to remove any traces of bleach; pat dry the embryos by placing the nylon mesh from the dechorionation chamber on a pile of Kleenex tissues for a few minutes (Figure 2F).
  9. Dechorionated embryos are hygroscopic in nature and tend to stick to each other. After pat drying for 2–3 min, gently pick up the clusters of embryos with a fine brush or dissecting needle; place them on a rectangular block of apple juice agar cut from the square cell culture plates containing a uniform layer of apple/grape juice agar medium (Figure 2G–H).
- **Embryo alignment and picking onto a coverslip**
  1. Align the embryos in a linear fashion on the edge of the rectangular agar block. Maintain the orientation of the embryos in such a way that the dorsal side faces the edge of the agar block, and the lateral side faces up to visualize the abdominal AMPs (Figure 2I). For other specific tissues or cells, the embryos can be aligned accordingly so they can be visualized easily using an inverted microscope.
  2. Place approximately 10 strips of double-sided tape (10 × 1 cm) in a 100 mL glass bottle and fill it with 90 mL of N-heptane. Allow the tape and heptane mixture to sit at room temperature for 12 h for proper extraction of the glue. The heptane/glue solution can be kept at 4 °C and used for several experiments.
  3. Using a P10 micropipette, add a small volume of heptane/glue solution to the center of a rectangular coverslip in a thin line that covers the entire coverslip length (Figure 2J).
  4. Allow the glue to semidry for 5 min in a sterile empty plastic box.
  5. Gently press the side containing glue against the aligned embryos on the agar block; this way, the embryos are transferred to the coverslip in the same alignment of the agar block, with the lateral side facing towards the coverslip (Figure 2K). This side can be easily visualized with high magnification lenses, which require applying immersion oil to the coverslip.
  6. Cover the embryos with a thin layer of halocarbon oil 27 to prevent them from desiccation (Figure 2L). Halocarbon oil allows embryos to exchange gases with the surrounding air, keeping them in healthy conditions for a long period.
- **Imaging the embryos with a confocal microscope using time-lapse series**
  1. Visualize the embryos using an inverted microscope, in which the objectives can touch the bottom side of the coverslip without disturbing the embryos (Figure 2M). Place the coverslips in the microscope in the same manner as glass slides using suitable holders.
  2. Using the lower magnification eyepiece of a Leica SP5 confocal microscope with time series imaging, select suitable embryos with appropriate developmental stage and better visualization of muscle stem cells and surrounding tissues (Figure 2N).
  3. Use the 40× oil immersion objective for enlargement of particular hemisegments of the embryo and better observation of the tissues of interest. Select Z-stacks based on the depth of tissue needed to be imaged (Figure 2O).
  4. Using the Fiji (ImageJ) software, analyze the raw files from the confocal microscope and generate images with the Z-stack of the selected optical section.
  5. Using Imaris (BitPlane) software, perform 3D reconstruction of both single time-point image and time-series live imaging.

## Data analysis

1. For maximum intensity projection of the single time-point image, select the desired optical sections in ImageJ and export them as a Z-stack picture.
2. Similarly, use the Imaris software to generate a 3D rotating model of either a single time-point projection or images captured on a time-lapse series mode.

**Video 1.**
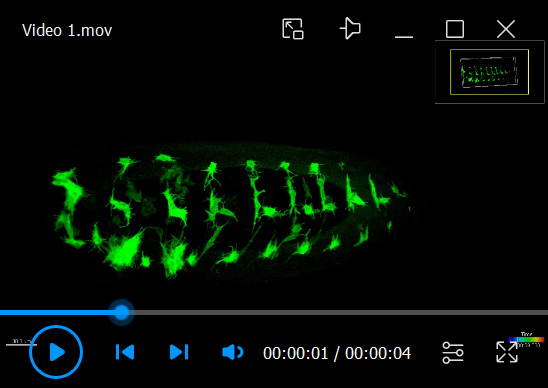
Time-lapse live imaging of M6-gapGFP embryos displaying dynamic behaviour of filopodia from the newly formed AMPs

## References

[1] Aradhya R., Zmojdzian M., Da Ponte J. P. and Jagla K.(2015). Muscle niche-driven Insulin-Notch-Myc cascade reactivates dormant Adult Muscle Precursors in *Drosophila*. Elife 4: e08497. 26650355 10.7554/eLife.08497 PMC4749548

[2] Bate M., Rushton E. and Currie D. A.(1991). Cells with persistent twist expression are the embryonic precursors of adult muscles in *Drosophila*. Development 113(1): 79-89. 1765010 10.1242/dev.113.1.79

[3] Boulina M., Samarajeewa H., Baker J. D., Kim M. D. and Chiba A.(2013). Live imaging of multicolor-labeled cells in *Drosophila*. Development 140(7): 1605-1613. 23482495 10.1242/dev.088930 PMC3596998

[4] Broadie K. S. and Bate M.(1991). The development of adult muscles in *Drosophila*: ablation of identified muscle precursor cells. Development 113(1): 103-118. 1764988 10.1242/dev.113.1.103

[5] Cold Spring Harb Protoc(2011). *Drosophila* apple juice-agar plates recipe. doi: 10.1101/pdb.rec065672

[6] Figeac N., Jagla T., Aradhya R., Da Ponte J. P. and Jagla K.(2010). *Drosophila* adult muscle precursors form a network of interconnected cells and are specified by the rhomboid-triggered EGF pathway. Development 137(12): 1965-1973. 20463031 10.1242/dev.049080

[7] Hadjieconomou D., Rotkopf S., Alexandre C., Bell D. M., Dickson B. J. and Salecker I.(2011). Flybow: genetic multicolor cell labeling for neural circuit analysis in *Drosophila melanogaster*. Nat Methods 8(3): 260-266. 21297619 10.1038/nmeth.1567

[8] Hampel S., Chung P., McKellar C. E., Hall D., Looger L. L. and Simpson J. H.(2011). *Drosophila* Brainbow: a recombinase-based fluorescence labeling technique to subdivide neural expression patterns. Nat Methods 8(3): 253-259. 21297621 10.1038/nmeth.1566 PMC3077945

[9] Sambasivan R. and Tajbakhsh S.(2015). Adult skeletal muscle stem cells. Results Probl Cell Differ 56: 191-213. 25344672 10.1007/978-3-662-44608-9_9

[10] Sellin J., Drechsler M., Nguyen H. T. and Paululat A.(2009). Antagonistic function of Lmd and Zfh1 fine tunes cell fate decisions in the Twi and Tin positive mesoderm of *Drosophila melanogaster*. Dev Biol 326(2): 444-455. 19028484 10.1016/j.ydbio.2008.10.041

